# New Treatment Options for MASLD Patients with Type 2 Diabetes

**DOI:** 10.3390/life16020254

**Published:** 2026-02-02

**Authors:** Andrea Mega, Chiara Turri, Luca Marzi, Marco Dauriz, Rodolfo Sacco, Annarosa Floreani, Cristina Stasi

**Affiliations:** 1 Department of Gastroenterology, South-Tyrolean Healthcare System (SABES-ASDAA), Bolzano General Hospital, 39100 Bolzano, Italy; chiara.turri@sabes.it (C.T.); luca.marzi@sabes.it (L.M.); 2 Section of Endocrinology and Diabetes, Department of Internal Medicine, South-Tyrolean Healthcare System (SABES-ASDAA), Bolzano General Hospital, 39100 Bolzano, Italy; marco.dauriz@sabes.it; 3 Gastroenterology and Digestive Endoscopy Unit, Department of Surgical and Medical Sciences, University of Foggia, Viale Pinto 1, 71122 Foggia, Italy; saccorodolfo@hotmail.com; 4 University of Padova, 35128 Padua, Italy; annarosa.floreani@unipd.it; 5 IRCCS Negrar, 37024 Verona, Italy; 6 Department of Life Science, Health and Health Professions, Link Campus University, 00165 Roma, Italy

**Keywords:** metabolic dysfunction-associated steatotic liver disease, metabolic dysfunction-associated steatohepatitis, fibrosis, cirrhosis, type 2 diabetes, oral glucose-lowering agent, transient elastography, glucagon-like peptide-1 agonists, sodium-glucose co-transporter 2 inhibitors

## Abstract

Metabolic dysfunction-associated steatotic liver disease (MASLD) is defined by hepatic steatosis in individuals with at least one cardiometabolic risk factor, most commonly type 2 diabetes mellitus (T2DM). People with non-alcoholic fatty liver disease, even without other metabolic factors, have a higher risk of T2DM. MASLD includes isolated liver steatosis, metabolic dysfunction-associated steatohepatitis, fibrosis, cirrhosis, and MASH-related hepatocellular carcinoma. MASLD patients are also at a higher risk of developing T2DM than the general population. International guidelines recommend a stepwise approach for identifying those at high risk of fibrotic progression, using the FIB-4 index for initial screening, followed by transient elastography. The link between MASLD and T2DM is notable due to shared pathophysiological mechanisms, some of which are reversible with treatment used in T2DM. Many new glucose-lowering drugs have also proven effective in improving anthropometric and metabolic parameters, as well as the stage of hepatic steatosis and fibrosis. Recent evidence suggests that GLP-1RAs and SGLT2is have beneficial effects in MASLD patients with T2DM. Specifically, GLP-1RAs improve hepatic insulin signaling, modulate lipid metabolism, reduce inflammation, and decrease hepatocyte oxidative stress. European guidelines recommend resmetirom as a MASH-targeted therapy, if locally approved, for adults with non-cirrhotic MASH and significant liver fibrosis (stage ≥ 2) and GLP-1RAs in MASH, including compensated cirrhosis, but they should be used for their respective indications, such as T2DM and obesity. Given the post-COVID burden of MASLD and its high risk of liver fibrosis progression among T2DM patients, this review specifically provides an overview of the complex relationship between MASLD and T2DM. Additionally, it examines current understanding of liver fibrosis evaluation and the effects of novel treatment options, with a particular focus on glucose-lowering therapies and their effects on necroinflammation, hepatic fat accumulation, and fibrosis progression in patients with MASLD and T2DM.

## 1. State-of-the-Art

In 1980, Ludwig and colleagues first described nonalcoholic steatohepatitis (NASH) as a progressive form of nonalcoholic fatty liver disease (NAFLD), which encompassed a spectrum of conditions from simple steatosis to steatohepatitis, fibrosis, cirrhosis, and hepatocellular carcinoma [1]. In 2020, the term NAFLD was reclassified as metabolic dysfunction-associated fatty liver disease (MAFLD) to emphasize the requirement for metabolic abnormalities [2]. In 2023, the terminology was further refined to MASLD and metabolic dysfunction-associated steatohepatitis (MASH), following a Delphi consensus process, to better reflect the central role of metabolic dysfunction and to avoid the stigmatizing language of “nonalcoholic” and “fatty” previously used in NAFLD [3,4,5].

MASLD is now defined as hepatic steatosis in combination with at least one cardiometabolic risk factor [5,6,7]. The presence of hepatic steatosis can be documented by several imaging methods such as ultrasound (simple and inexpensive, but with reduced sensitivity when steatosis is <20%) [8]; controlled attenuation parameter (CAP) using elastography (FibroScan) that allows semi-quantitative quantification of steatosis [9]; and magnetic resonance imaging (in particular MRI-proton density fat fraction, PDFF), which represents the most accurate method for quantifying hepatic lipid content, even in clinical studies [10,11]. In addition, hepatic steatosis can be identified by serum biomarkers or indirect scores (e.g., fatty liver index, FLI) [12,13] and directly by histology (liver biopsy). The second diagnostic criterion includes the presence of at least one criterion of metabolic dysfunction and specifically the presence of obesity or overweight (BMI ≥ 25 kg/m^2^, ≥23 kg/m^2^ for Asian populations), type 2 diabetes mellitus (T2DM), or at least two factors of metabolic dysfunction (among hypertension, dyslipidemia, insulin resistance, increased waist circumference). Finally, it is essential to rule out other causes of steatosis and chronic liver disease, including alcohol abuse (consumption > 30 g/day in men, >20 g/day in women), chronic viral hepatitis (HBV and HCV), autoimmune liver diseases, steatogenic drugs (amiodarone, tamoxifen, methotrexate, corticosteroids, certain antivirals), hereditary metabolic diseases (e.g., Wilson’s disease or alpha-1-antitrypsin deficiency) [14,15].

The diagnostic process emphasizes the identification of metabolic risk rather than exclusion of other causes and recognizes the new category of MetALD for cases with overlapping metabolic and alcohol-related liver injury [2,3,6]. Type 2 diabetes mellitus is one of the key diagnostic criteria for MASLD because its presence accelerates the progression of liver disease towards fibrosis, cirrhosis, and hepatocellular carcinoma. In fact, the European Association for the Study of the Liver, the European Association for the Study of Diabetes, and the European Association for the Study of Obesity (EASL–EASD–EASO) guidelines [15] recommend active monitoring of liver function in all patients with T2DM.

The American Diabetes Association [16] defines T2DM as a condition in which insulin resistance and impaired β-cell function lead to persistent hyperglycemia. Most individuals are asymptomatic at diagnosis, but classic symptoms may include polyuria, polydipsia, fatigue, blurred vision, and unintentional weight loss [16,17,18].

Diagnostic criteria for T2DM, as established by the American Diabetes Association [16] and the World Health Organization [19], require one of the following in a nonpregnant adult: fasting plasma glucose ≥ 126 mg/dL (≥7.0 mmol/L) after at least 8 h of fasting; hemoglobin HbA1C ≥ 6.5% (≥48 mmol/mol) using a standardized assay 2 h plasma glucose ≥ 200 mg/dL (≥11.1 mmol/L) during a 75 g oral glucose tolerance test; and random plasma glucose ≥ 200 mg/dL (≥11.1 mmol/L) in the presence of classic symptoms of hyperglycemia or hyperglycemic crisis.

In the absence of unequivocal hyperglycemia, diagnosis should be confirmed by repeat testing with the same or a different test [17,18,19,20].

A recent meta-analysis [14] reported a global pooled period prevalence of MASLD among patients with T2DM of 65%, rising to 69% in 2021. The meta-analysis included 123 studies involving 2,241,753 patients with T2DM, with a mean age of 58.7 years (range, 44.8–74 years). Several studies demonstrated a significant increase in the global burden of MASLD in the post-COVID era [21]; moreover, the prevalence of MASLD among patients with T2DM is considerable. Large-scale meta-analyses report that approximately two-thirds of individuals with T2DM exhibit hepatic steatosis, while up to about 20% present with advanced fibrosis (F3–F4) [14]. Conversely, individuals with MASLD face a two- to five-fold higher risk of developing T2DM than the general population, largely attributable to shared risk factors and systemic insulin resistance [22]. Type 2 diabetes mellitus is a chronic metabolic disorder characterized by hyperglycemia resulting from a combination of insulin resistance and a relative insulin deficiency. It accounts for 90–95% of all diabetes cases globally, with current estimates indicating a worldwide prevalence between 589 and 828 million adults and rising incidence in regions with increasing rates of obesity and sedentary lifestyle [17,23]. The disease is associated with significant morbidity and mortality due to its complications, including cardiovascular disease, kidney failure, retinopathy, and neuropathy [17,23].

Based on these premises and the increasing global prevalence of MASLD and its high risk of progression to hepatic fibrosis among patients with T2DM, this review focuses on the relationship between MASLD and T2DM with a particular emphasis on liver fibrosis evaluation and the effects of novel therapeutic options, focusing on glucose-lowering agents, such as glucagon-like peptide-1 receptor agonists (GLP-1RAs) and sodium–glucose co-transporter inhibitors (SGLT2is), on hepatic necroinflammation, fat accumulation, and fibrosis progression in patients with MASLD and T2DM.

## 2. Pathogenesis of MASLD and Its Relationship with Type 2 Diabetes Mellitus

Several epidemiological studies reported a high risk of T2DM in patients with steatosis [22]. Cho et al. [24], in a study of 5439 patients with steatosis without metabolic dysregulation who did not meet MASLD criteria, reported an elevated risk of developing T2DM. Mantovani et al. [22] demonstrated that the risk increased with the severity of fibrosis and steatosis, supporting a role for MASLD in the development of T2DM.

Excess free fatty acids induce oxidative stress by producing reactive oxygen species (ROS), leading to oxidative injury to lipids, proteins, and DNA. Mitochondrial dysfunction further impairs fatty acid oxidation, exacerbating this vicious cycle. Moreover, ROS promote lipid peroxidation and activate cell death pathways (apoptosis and necroptosis) [25].

Alterations in gut microbiota composition and function (dysbiosis) also contribute to MASLD pathogenesis. Key mechanisms include increased intestinal permeability and bacterial translocation, lipopolysaccharide (LPS)-driven endotoxemia, activation of Toll-like receptors (TLR4), and systemic inflammatory responses. Additionally, microbiota-derived metabolites (short-chain fatty acids, secondary bile acids, and endogenous ethanol) directly modulate hepatic metabolism and immune responses [26,27].

Lipotoxins and endotoxemia activate Kupffer cells and other resident immune cells, triggering the release of pro-inflammatory cytokines (TNF-α, IL-6, and IL-1β). This cascade amplifies hepatocellular injury and promotes progression to MASH [7,25].

Hepatocyte death and the ensuing inflammatory response lead to the release of pro-fibrogenic mediators that activate hepatic stellate cells, transforming them into myofibroblasts responsible for extracellular matrix production. The outcome is progressive collagen deposition and fibrosis development. Fibrosis, more than steatosis or isolated inflammation, represents the key prognostic determinant of the disease [28].

Several genetic variants and epigenetic modifications modulate individual susceptibility [29,30].

The relationship between MASLD and T2DM is bidirectional and complex, with significant pathogenetic, clinical, and prognostic implications [7,15,31].

In MASLD, intrahepatic lipid accumulation interferes with insulin signaling, resulting in impaired suppression of gluconeogenesis and fasting hyperglycemia [32]. In turn, the chronic hyperglycemia and compensatory hyperinsulinemia characteristic of diabetes exacerbate hepatic de novo lipogenesis and lipid deposition, thereby perpetuating a vicious cycle [7,33].

Hepatic steatosis also activates pro-inflammatory pathways and promotes cytokine release (TNF-α, IL-6, and MCP-1), which contribute to peripheral insulin resistance, endothelial dysfunction, and progression of atherosclerosis [34]. Moreover, lipotoxic species, such as ceramides and diacylglycerols, impair insulin receptor phosphorylation in muscle and adipose tissue, thereby amplifying metabolic dysfunction [32,33].

The coexistence of MASLD and T2DM substantially increases the risk of both hepatic and systemic complications. In diabetic patients, the progression from MASLD to MASH and advanced fibrosis occurs more rapidly, with a higher risk of cirrhosis and hepatocellular carcinoma (HCC) [35]. Extrahepatically, patients with MASLD and T2DM are at greater risk of major adverse cardiovascular events (MACE) and chronic kidney disease [7,34].

Owing to these associations, the EASL–EASD–EASO [15] and American Diabetes Association/European Association for the Study of Diabetes (ADA/EASD) [35] recommend systematic evaluation for MASLD in patients with T2DM, as this population represents the highest-risk group for complications and constitutes a priority target for secondary prevention. This approach requires integrated, multidisciplinary strategies for diagnosis, monitoring, and therapeutic management [31,36].

## 3. Assessment of Hepatic Fibrosis in MASLD

As mentioned above, the diagnostic algorithm outlined in the guidelines [15] follows a stepwise approach. The first step is to identify patients at risk (T2DM, obesity, metabolic syndrome). Next, an initial screening using non-invasive scores such as Fibrosis-4 (FIB-4) and Nonalcoholic Fatty Liver Disease Fibrosis Score (NFS) is performed. If these results are indeterminate or indicate high risk, the next step is to perform further investigation with elastography, such as VCTE and MRE. Finally, in selected situations, a liver biopsy may be considered, as it remains the gold standard for confirming a diagnosis of MASH and accurately assessing the stage of fibrosis, particularly when non-invasive tests yield conflicting results, when suspicion of concomitant liver disease is high, or when enrolling in a clinical trial [37].

This sequential algorithm is justified by the need to efficiently identify patients at the highest risk for adverse liver outcomes, particularly in populations with T2DM or multiple metabolic risk factors. The rationale is to avoid unnecessary specialist referral and invasive procedures in low-risk patients while ensuring timely evaluation and intervention for those at higher risk [38].

One of the main non-invasive serological scores recommended for estimating the stage of liver fibrosis is the FIB-4 index, which is recommended as a first-line screening tool due to its accessibility, low cost, and robust negative predictive value for excluding advanced fibrosis (≥F3–F4) [6,18]. In patients aged 65 years or younger, FIB-4 values < 1.3 indicate a low likelihood of advanced fibrosis, values between 1.3 and 2.67 are considered indeterminate, and values above 2.67 suggest a high probability of advanced fibrosis. For patients with indeterminate FIB-4 values, further risk stratification is recommended using a second-line non-invasive test, such as VCTE or an alternative serum-based fibrosis score [6]. In cases of discordant results, liver biopsy is advised [5,15]. Furthermore, studies by McPherson et al. [39] and Sung et al. [40] have established age-specific FIB-4 cutoffs for older adults, indicating that a threshold of approximately 2.0 in individuals aged 65 years or older enhances diagnostic specificity and prediction of advanced fibrosis compared to the conventional cutoff of 2.67.

The AST-to-c (APRI) is another widely available noninvasive score that can help rule out advanced fibrosis, but its diagnostic accuracy is generally inferior to the FIB-4 index. APRI has moderate diagnostic accuracy for advanced fibrosis, but it is less reliable than the FIB-4 and the Nonalcoholic Fatty Liver Disease Fibrosis Score (NFS), especially in patients with pure metabolic etiologies or in pediatric populations [41,42,43,44]; the Nonalcoholic Fatty Liver Disease Fibrosis Score incorporates age, BMI, hyperglycemia, AST/ALT ratio, platelet count, and albumin. It performs similarly to FIB-4 for advanced fibrosis in MASLD/MASH patients, with high specificity at upper cutoffs but a large indeterminate range [13,45].

Non-invasive methods play a key role in assessing liver fibrosis in patients with MASLD (Table 1).

The American Diabetes Association [6] recommends vibration-controlled transient elastography (VCTE, e.g., FibroScan^®^) as the best-validated imaging technique for fibrosis risk stratification in MASLD. VCTE measures the speed of propagation of mechanical waves generated through the liver to obtain the “liver stiffness measurement” (LSM), with liver stiffness values (LSV) < 8.0 kPa having a high negative predictive value for excluding advanced fibrosis (≥F3–F4), and values ≥ 8.0 kPa indicating increased risk and warranting referral to hepatology [6]. Stiffness threshold values (kPa) must be interpreted in the clinical context. Thresholds vary depending on the population studied (MASLD, viral hepatitis, alcohol-related liver disease, cholestatic liver disease, as summarized in Table 2).

Moreover, in the MASLD population, the LSV cut-off varies depending on the probe type (M vs. XL, as summarized in Table 3), BMI, the presence of acute inflammation or cholestasis, and the purpose of the examination (rule-out vs. rule-in).

Several studies have demonstrated key limitations that affect test interpretation, including acute inflammation [58,59], alcohol intake, congestive hepatopathy [60], and BMI/probe choice for VCTE [61]. Several studies have demonstrated that VCTE measurements correlate significantly with the METAVIR scoring system.

The METAVIR system itself represents a well-established histopathological framework designed to evaluate both the degree of necro-inflammatory activity and the stage of hepatic fibrosis in biopsy samples from patients with chronic hepatitis (F0 = absence of fibrosis, F1 = portal fibrosis without intralobular septa, F2 = portal fibrosis with rare intralobular septa, F3 = portal fibrosis with intralobular septa/occasional nodules, F4 = cirrhosis [62]). Fibrosis staging is often expressed using METAVIR categories to ensure comparability with previous studies and validation cohorts for non-invasive tests, although different histological scoring systems are used to assess MASLD/MASH, such as MASH CRN scoring [63,64]. While liver biopsy remains the reference standard, the consistent association between VCTE values and METAVIR stages makes VCTE preferable due to its accessibility, rapid results, and strong validation against histology [6,18].

Magnetic resonance imaging, particularly MRE, offers higher diagnostic accuracy for staging fibrosis and is recommended in specialty clinics when VCTE is inconclusive or unreliable. MRE-derived LSV > 3.5 kPa suggests advanced fibrosis, and >4.4 kPa is consistent with cirrhosis. MRI-derived iron-corrected T1 (cT1) can also help identify at-risk MASH. However, MRI/MRE is limited by cost and availability, so it is not recommended for initial risk stratification in general practice [6].

MRI-based techniques, including T1 mapping and extracellular volume quantification, are emerging as alternative noninvasive methods for fibrosis staging, demonstrating strong correlation with MRE and histology [65]. However, their use is currently limited to research and tertiary care due to cost and availability.

## 4. Focus on Therapy

### 4.1. Drug Therapy for Type 2 Diabetes Mellitus

The pathogenesis of MASLD is characterized by marked heterogeneity and complex metabolic-inflammatory interactions. As a result, targeted therapies are limited and require personalized treatment strategies based on cardiometabolic comorbidities. The management of T2DM has undergone profound changes in recent years, with an increasing focus on the prevention of cardiovascular, renal, and hepatic complications. The ADA/EASD guidelines [35] emphasize the importance of a personalized approach that considers not only glycemic control but also overall cardiovascular risk and the presence of MASLD [7,31].

The therapeutic goals of the drug in T2DM are

- Reduction in blood glucose levels and maintenance of hemoglobin A1c (HbA1c) < 7% (with individualized targets based on age, comorbidities, and hypoglycemia risk).
- Prevention of microvascular and macrovascular complications.
- Body weight control and improvement of the metabolic profile.
- Organ protection (heart, kidney, liver) [31].

Treatment strategies for T2DM include metformin, sulfonylureas, thiazolidinediones, dipeptidyl peptidase-4 (DPP-4) inhibitors, SGLT2i, GLP-1RA, dual gastric inhibitory polypeptide and glucagon-like peptide-1 (GIP/GLP-1) receptor agonists, and insulin [15,66,67,68,69]. A meta-analysis [66], involving 307 subjects without T2DM from six RCTs, demonstrated that metformin significantly reduced BMI and AST, underscoring the clinical importance of metformin administration to improve liver function and body composition in non-diabetic MASLD patients. The American College of Physicians recommends metformin as first-line therapy for most patients, with SGLT2i or GLP-1RA prioritized in those with cardiovascular, renal, or heart failure comorbidities [17,35,69,70,71,72,73].

### 4.2. Non-Pharmacological Therapies in MASLD

Non-pharmacological interventions represent the cornerstone of treatment for MASLD and are recommended as first-line strategies in all major international guidelines [7,15,35,74].

Weight loss is the principal determinant of improvement in steatosis, inflammation, and fibrosis: a reduction of ≥5% in body weight improves steatosis; a reduction of ≥7–10% is associated with resolution of MASH and fibrosis reduction [15].

Weight loss can be achieved through dietary interventions, physical activity, or bariatric surgery in patients with severe obesity. No single dietary pattern is universally recommended, but clinical studies have demonstrated benefits from the Mediterranean diet (rich in fruits, vegetables, legumes, whole grains, fish, and extra-virgin olive oil; improves insulin sensitivity and reduces intrahepatic fat) [75], hypocaloric diets, and regimens with reduced intake of simple sugars and fructose.

Regular physical activity improves insulin sensitivity, reduces intrahepatic fat, and enhances mitochondrial function. At least 150–200 min of moderate-intensity aerobic activity per week (e.g., brisk walking, cycling, swimming) is recommended. The benefits are greater when aerobic exercise is combined with resistance training [15,76].

In addition to diet and regular physical activity, other lifestyle interventions with significant metabolic impact include abstinence from or marked reduction in alcohol consumption, limiting saturated fat and processed meat intake, and improving sleep quality while reducing stress [77].

Bariatric surgery is indicated in patients with severe obesity (BMI ≥ 40, or ≥35 with comorbidities) who do not achieve adequate results with conservative approaches. Among surgical procedures, gastric bypass and sleeve gastrectomy are the most effective [78].

### 4.3. Pharmacological Treatment of MASLD

Lifestyle modification remains the cornerstone of therapy for MASLD. Sustained weight reduction of at least 7–10% through dietary modification, regular physical activity, and behavioral interventions is consistently associated with histological improvement in steatosis, inflammation, and, in some cases, fibrosis.

Bariatric surgery and structured programs for weight loss may be considered in selected patients, but pharmacological therapy should always be viewed as adjunctive to lifestyle intervention rather than as first-line management [7,31,78,79,80,81].

Currently, several agents originally developed for T2DM or obesity management demonstrate hepatic benefits and are endorsed by international societies. In addition, treatment with resmetirom, in the countries where it has been approved, may be considered for individuals with MASLD who are non-cirrhotic and have advanced fibrosis or steatohepatitis at risk of progression to significant fibrosis, assessed when available by liver biopsy, or validated non-invasive methods, or in patients at increased risk for liver-related adverse outcomes [15]. As strongly recommended by EASL–EASD–EASO guidelines [15], GLP1RAs should be used for their respective indications (T2DM and obesity) and are safe in compensated cirrhosis-MASH-related, and they also improve cardiometabolic outcomes. Similarly, SGLT2 inhibitors are not recommended as MASH-targeted therapies but should be used for their respective indications, namely, T2DM, heart failure, and chronic kidney disease [15]. Evidence suggests that pioglitazone treatment can improve steatohepatitis and insulin sensitivity, and in long-term evaluation, liver fibrosis [82]. The ADA recommends pioglitazone, a GLP-1RA, or a dual GLP-1RA as preferred glycemic management for adults with T2DM and biopsy-confirmed MASH, or those at high risk of liver fibrosis based on noninvasive assessment, because of their potential benefits for MASH [83]. Pioglitazone addresses multiple aspects of MASLD, improves cardiovascular outcomes, and is effective for MASH. Although side effects are a concern, they can be reduced by optimizing dosing and combining pioglitazone with other medications such as metformin, SGLT2 inhibitors, or GLP-1 RAs [84].

The therapeutic choice should be individualized according to comorbidities, risk-benefit profile, and local availability.

Table 4 summarizes the main therapies currently available, listing their mechanisms of action, scientific evidence, indications, and contraindications.

## 5. Summary of Clinical Evidence on GLP-1RA and SGLT2i in MASLD

Assuming that pharmacotherapy should be used selectively, in combination with lifestyle interventions, and tailored to comorbid conditions, safety profiles, and patient preferences, international guidelines [15,16,35] emphasize prioritizing GLP-1RA and SGLT2i in MASLD patients with T2DM or obesity, due to their dual metabolic and hepatic benefits.

### 5.1. Methods

A structured narrative review was conducted to explore new glucose-lowering treatment options for patients with MASLD and T2DM. A comprehensive search of PubMed databases was performed up to 2 January 2026. The randomized clinical trial (RCT) was eligible for inclusion if it evaluated sodium–glucose co-transporter 2 inhibitors (empagliflozin and dapagliflozin) or glucagon-like peptide receptor agonists considered safe in MASLD and should be used for their respective indications (e.g., T2DM) by guidelines, with some also approved for obesity (semaglutide, tirzepatide, and also liraglutide, as a dual GIP/GLP-1 RA) [15], and also evaluated liver fibrosis. RCTs with combination treatments were excluded.

Search terms combined the following keywords and Boolean operators: “glucagon like peptide receptor agonists and MASLD and fibrosis” (first item) “glucagon like peptide receptor agonists and MAFLD and fibrosis” (second item), “glucagon like peptide receptor agonists and NAFLD and fibrosis” (third item), “glucagon like peptide-1 receptor agonists and non-alcoholic steatohepatitis and fibrosis” (fourth item), “sodium-glucose co-transporter 2 inhibitors and MASLD and fibrosis” (fifth item), “sodium-glucose co-transporter 2 inhibitors and MAFLD and fibrosis” (sixth item), “sodium-glucose co-transporter 2 inhibitors and NAFLD and fibrosis” (seventh item), “sodium-glucose co-transporter 2 inhibitors and non-alcoholic steatohepatitis and fibrosis” (eighth item). No time or language restrictions were initially applied; however, studies without an English version were excluded. Conference abstracts, letters, posters, and gray literature were excluded. Case reports and case series were classified as low quality by default. Exclusion study designs included reviews, clinical guidelines, expert consensus statements, editor’s letters, preclinical studies, and pediatric and adolescent studies. Two independent reviewers screened titles and abstracts and then reviewed the full-text articles. A third reviewer participated by screening titles, abstracts, and full-text articles and by helping resolve disagreements through discussion.

**Figure 1:**
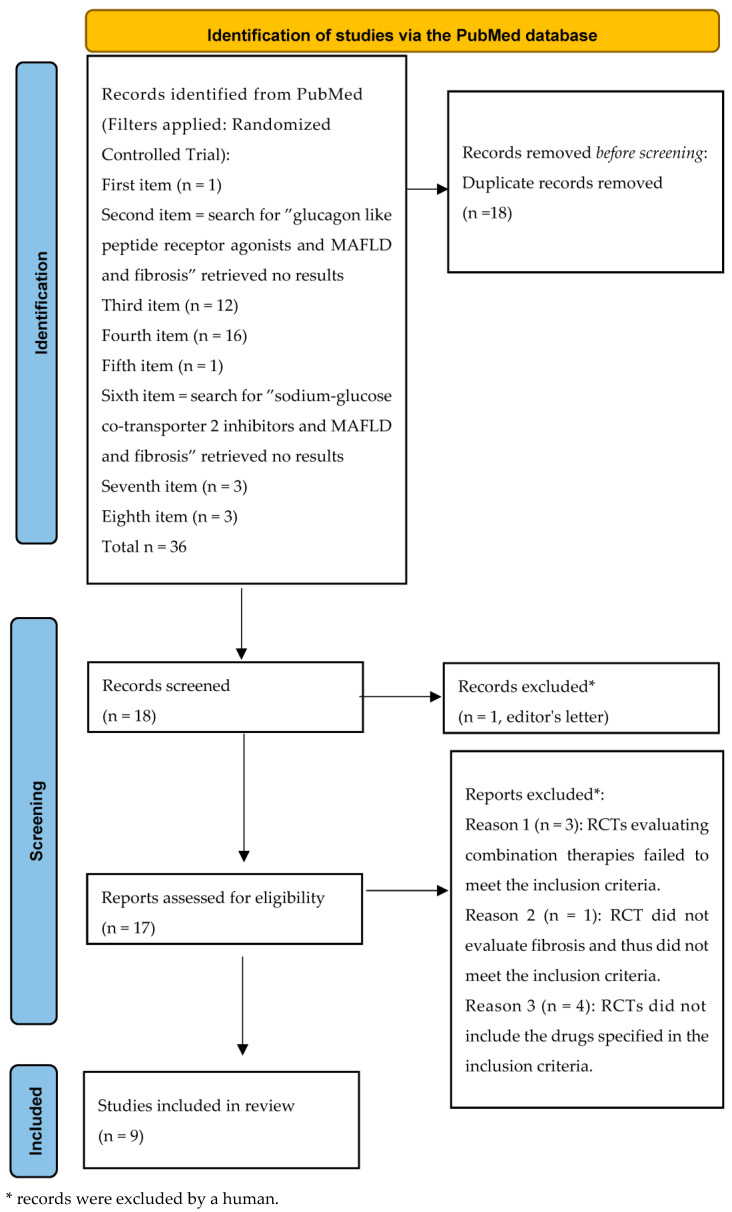
Flowchart of the randomized clinical trial research.

Duplicates were removed (*n* = 18). Eighteen unique RCTs remained. Of these, nine met the inclusion criteria (Figure 1, like the PRISMA style [104]).

This review has some limitations. As a narrative review, it did not include a formal assessment of methodological quality or risk of bias of the included studies. Therefore, study selection and interpretation may be subject to author evaluation, increasing the risk for selection bias. Moreover, heterogeneity in study design and in population characteristics (genetics, ethnicity, or socioeconomic status) limits direct comparisons across studies.

### 5.2. Randomized Clinical Trials Investigating GLP-1RA, SGLT2i, and GIP/GLP-1 RA in MASLD

Table 5 summarizes the main randomized clinical trials investigating the efficacy of GLP-1RA, SGLT2i, and GIP/GLP-1 RA in the treatment of MASH and fibrosis in MASLD patients.

The LEAN study [88] randomly assigned 52 patients to receive either liraglutide (*n* = 26) or placebo (*n* = 26). The primary outcome was assessed by histological evaluation, measuring improvement from baseline to 48 weeks, defined as resolution of steatohepatitis indicated by the disappearance of hepatocyte swelling without worsening of fibrosis. Secondary histological outcomes included changes in the overall MASLD activity score, which comprises steatosis, hepatocyte swelling, and lobular inflammation, as well as the Kleiner fibrosis stage. At baseline, 38% of patients in the liraglutide group and 42% in the placebo group had Kleiner fibrosis stage 3, while cirrhosis was present in 8% of patients treated with liraglutide and 15% in the placebo group. After treatment, the liraglutide group showed a lower rate of fibrosis progression and a higher proportion of improvements in steatosis than the placebo group. When stratified by diabetes status and trial site, the odds ratio for treatment effect was 7.8. Fewer patients experienced worsening fibrosis with liraglutide (9%) than with placebo (36%). However, liraglutide did not significantly alter the composite MASLD activity score. The lack of difference in mean change in fibrosis stage between groups likely reflects the treatment duration, suggesting that a longer course should be evaluated. A similar endpoint was employed in the phase 2 study by Newsome et al. [89], which included patients with biopsy-proven MASH and liver fibrosis stage F1, F2, or F3 (Kleiner fibrosis score). Participants were randomly assigned to receive semaglutide once daily at doses of 0.1 mg (80 patients), 0.2 mg (78 patients), or 0.4 mg (82 patients) or placebo (80 patients). Among those with fibrosis stage F2 or F3, the proportion achieving resolution of NASH without worsening of fibrosis after 72 weeks was significantly higher in the semaglutide groups, with the greatest effect in the 0.4 mg group (59% versus 17% in the placebo group). However, semaglutide did not result in a significant difference between groups regarding improvement of at least one fibrosis stage without worsening of MASH. Fibrosis progression occurred in 5% of patients in the semaglutide 0.4 mg group compared to 19% in the placebo group. The most recent phase 3 study by Sanyal et al. [105] was a multicenter, randomized, double-blind, placebo-controlled trial involving 1197 patients with biopsy-confirmed MASH and stage 2 or 3 fibrosis. Participants were randomized in a 2:1 ratio to receive either semaglutide 2.4 mg subcutaneously once weekly or placebo for 240 weeks. An interim analysis was performed on the first 800 patients at week 72. The primary endpoints were resolution of steatohepatitis without worsening of liver fibrosis and reduction in liver fibrosis without worsening of steatohepatitis. The results of this phase 3 trial demonstrate a significant resolution of steatohepatitis without worsening of fibrosis in 62.9% of the 534 patients receiving semaglutide, compared to 34.3% of the 266 patients receiving placebo. Additionally, a significant reduction in liver fibrosis without worsening of steatohepatitis was observed in 36.8% of patients in the semaglutide group and in 22.4% of patients in the placebo group.

The randomized, placebo-controlled study by Ratziu et al. [106] compared histologic assessment of MASH between pathologists and a machine-learning model trained on data from 251 patients with biopsy-proven MASH and fibrosis stage F1-F3 treated with subcutaneous semaglutide 0.1, 0.2, or 0.4 mg once daily for 72 weeks. Both methods found a significantly greater percentage of patients achieving the primary endpoint of MASH resolution without worsening fibrosis with semaglutide 0.4 mg compared with placebo. Continuous machine-learning-based scores demonstrated an antifibrotic effect not measured by conventional histopathology. Loomba et al. [107] investigated the effects of the randomly assigned (2:1) once-weekly subcutaneous semaglutide 2.4 mg compared with a visually matching placebo in patients with MASH and compensated cirrhosis. After 48 weeks, there was no statistically significant difference between the two groups in the proportion of patients with improvement in liver fibrosis of stage 1 or more without worsening of MASH, or in the proportion of patients who achieved MASH resolution. Caussy et al. [108] investigated the relationship between metabolic and histological responses in 190 patients randomly assigned to treatment with once-weekly tirzepatide 5 mg (*n* = 47), 10 mg (*n* = 47), 15 mg (*n* = 48), or placebo (*n* = 48) with fibrotic MASH. Overall, there were 80 responders (52%) (tirzepatide, *n* = 76; placebo, *n* = 4) and 74 (48%) nonresponders (tirzepatide, *n* = 44; placebo, *n* = 30) for the primary outcome of MASH resolution without worsening of fibrosis. Reductions in body weight were significantly correlated with reductions in hepatic fat content (assessed by MRI-PDFF), liver fibro-inflammation (assessed by MRI-cT1), MASH disease activity (assessed by NAS score), and improvements in fibrosis biomarkers, including ELF, PRO-C3, NIS4, and liver stiffness (assessed by VCTE). 

The majority of RCTs conducted to date, especially those assessing liver fibrosis stage via liver biopsy, have demonstrated resolution of MASH, but the RCT with Tirzepide appears to offer a more pronounced improvement in liver fibrosis.

Taheri et al. [110] conducted a prospective RCT in patients with MASLD without T2DM to evaluate the effects of empagliflozin on liver steatosis and fibrosis. The findings demonstrated no significant differences in CAP scores between the two groups, whereas significant differences in LVS were observed between the empagliflozin and placebo groups, with LVS decreasing significantly in the empagliflozin group. However, after 24 weeks, AST and ALT levels in the empagliflozin group had also significantly decreased, while no significant changes were observed in the placebo group. This could likely also influence LSV. Shimizu et al. [111] conducted a randomized, active-controlled, open-label trial investigating the effects of dapagliflozin on liver steatosis and fibrosis in 57 patients with T2DM and MASLD. Patients were randomly assigned to a dapagliflozin group (5 mg/d; *n* = 33) or a control group (*n* = 24), and treated for 24 weeks. The dapagliflozin group showed a significant reduction in CAP score (from 314 ± 61 to 290 ± 73 dB/m), whereas the control group showed no significant change. In 14 patients in the dapagliflozin group with LSM ≥ 8.0 kPa, LSV decreased significantly from 14.7 ± 5.7 to 11.0 ± 7.3 kPa. Lin et al. [112] conducted an RCT involving 78 MASH patients. The study demonstrated MASH improvement without worsening of fibrosis in 53% of patients in the dapagliflozin group and 30% in the placebo group, as well as MASH resolution without worsening of fibrosis in 23% of patients in the dapagliflozin group and 8% in the placebo group [112]. The trial also reported improvement in fibrosis without worsening of MASH [112].

Outside our search strategy for RCTs in our review, we report the results of the RCT by Kuchay et al. [91], which demonstrated a significant reduction in hepatic fat content in patients treated with empagliflozin (10 mg daily) compared to control after 20 weeks of treatment and assessed by MRI-PDFF. Accordingly, Albdegani et al. [113] examined the effect of empagliflozin on liver fat content in individuals with and without T2DM, demonstrating that empagliflozin reduced liver fat content, whereas the placebo group showed an increase. The decrease in liver fat was comparable in individuals with T2DM and those without and was strongly correlated with baseline liver fat content, decreased body weight, and improved insulin sensitivity.

A recent meta-analysis [114] showed that GLP-1RA is most effective for MASH; SGLT2i is beneficial for steatosis and metabolism; however, the effect on fibrosis remains uncertain. Overall, clinical studies conducted to date demonstrate that GLP-1RA induces high rates of MASH resolution, depending on the trial, with additional benefits on weight reduction [15].

Conversely, SGLT2i produces more modest yet consistent reductions in hepatic lipid content (−1.5% to −3.0% by MRI-PDFF) and confers robust cardiovascular and renal benefits [114]. Available meta-analyses confirm a favorable effect of both drug classes on steatosis and metabolic indices, while their antifibrotic impact remains uncertain and requires further long-term histological trials.

### 5.3. Future Directions

Recent research is focused on finding genetic variants that can predict patient responses to new diabetes therapies. The largest genome-wide study to date found that certain genetic variants (GLP1R rs6923761 and ARRB1 rs140226575) are linked to greater HbA1c reduction after GLP-1RA treatment in some groups, but these effects are small, and the variants are rare; genetic testing is not yet used in standard clinical care [115,116,117,118]. Studies confirm the association between GLP1R variants and glycemic response [119,120,121,122], but MASLD-specific prediction data are not yet available

For SGLT2i, most studies have concentrated on the SLC5A2 gene, which encodes the SGLT2 transporter. The rs9934336 polymorphism has been linked to variability in glycemic response and cardiovascular risk, although its clinical effect on SGLT2i response (e.g., to empagliflozin) appears modest and not consistently significant [115,121,122]. Polymorphisms in the UGT1A9 gene, which is involved in SGLT2i metabolism, have also been investigated, but the studies did not possess a clinically relevant impact [123].

## 6. Practical Clinical Consideration

This section presents clinical “how-to” content, emphasizing actionable steps.

**Figure 2:**
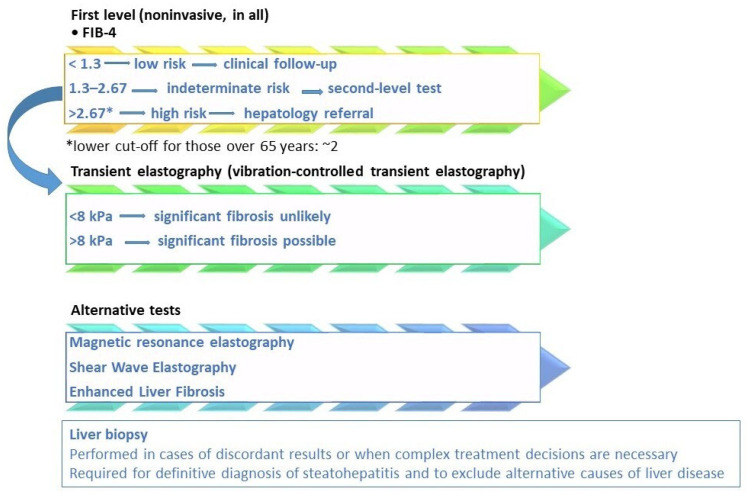
MASLD fibrosis risk stratification based on current guidelines and scientific evidence. Legend: the diagnostic algorithm outlined in the EASL–EASD–EASO Clinical Practice Guidelines [15] employs a stepwise approach to stratify fibrosis risk. Initial screening is conducted using the Fibrosis-4 (FIB-4) score. If results are indeterminate for advanced fibrosis (FIB-4 = 1.3–2.67) or suggest high risk (>2.67), further assessment with elastography, such as vibration-controlled transient elastography (VCTE), is recommended. To assess the high risk of fibrosis, lower FIB-4 cut-offs (approximately 2) should be considered in subjects over than 65 years [6,39,40]. The LSM cut-off of 8 kPa in clinical practice to exclude advanced fibrosis is the most validated, with an NPV greater than 90% [6,31]. Additional non-invasive tests, including magnetic resonance elastography (MRE) and the enhanced liver fibrosis (ELF) score, may also be utilized. In specific cases, liver biopsy may be warranted. Liver biopsy remains the definitive method for confirming a diagnosis of metabolic dysfunction-associated steatohepatitis (MASH) and for accurately staging fibrosis, particularly when non-invasive tests provide conflicting results, there is a high suspicion of concomitant liver disease, or for clinical trial eligibility [31].

Figure 2 shows MASLD fibrosis risk stratification per current guidelines. In fact, the choice of therapy must integrate the evaluation of fibrotic risk, metabolic comorbidities, liver stage, and long-term safety. Stefan et al. [124] identified three MASLD patient phenotypes: those with a hepatic genetic component, who face a higher risk of MASH and fibrosis; those with a metabolic component linked to hepatic de novo lipogenesis; and those with a metabolic component related to adipose tissue dysfunction. The latter two phenotypes carry a moderately increased risk of MASH and fibrosis.

Given the high prevalence of MASLD in people with obesity and T2DM, Nowak et al. [125] emphasized that tirzepatide and semaglutide may significantly reduce liver fat, improve liver biomarkers, and lower cardiometabolic risks.

**Figure 3:**
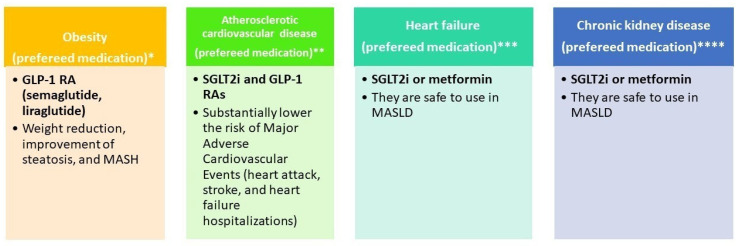
Glucose-lowering therapy by phenotype. * References: EASL–EASD–EASO [15]; Dusilová, T. et al. [126]; Xie, W. et al. [127]; ** References: Cusi et al. [6]; Zelniker, T. A. et al. [128]; *** References: EASL–EASD–EASO [15]; **** References: EASL–EASD–EASO [15].

Figure 3 illustrates the selection of glucose-lowering therapy based on metabolic comorbidities (obesity, atherosclerotic cardiovascular disease, heart failure, and chronic kidney disease).

For glucose-lowering therapy in MASLD cirrhosis, EASL–EASD–EASO guidelines [15] indicate that GLP-1RAs are safe for use in MASH, including compensated cirrhosis, and should be prescribed according to their approved indications. Metformin is appropriate for adults with compensated cirrhosis and preserved renal function, but it is contraindicated in adults with decompensated cirrhosis, particularly when renal impairment is present, due to the risk of lactic acidosis. GLP-1RAs are suitable for adults with Child–Pugh class A cirrhosis, while SGLT2 inhibitors may be used in adults with Child–Pugh class A and B cirrhosis. In a recent large nationwide cohort study including 22,550 patients with MASLD [129], SGLT-2i was associated with a lower risk of hepatic decompensation events compared with thiazolidinediones, while demonstrating similar effectiveness to GLP-1RA. Therefore, GLP-1 RAs are promising for the management of MASLD, as they support weight loss, improve glycemic control, and reduce liver inflammation [125].

## 7. Conclusions

Non-invasive methods are essential for evaluating fat accumulation and liver fibrosis. However, liver biopsy remains the most reliable tool for detecting necroinflammation, steatosis, and fibrosis, particularly when non-invasive assessments yield conflicting results, there is suspicion of concomitant liver disease, or clinical trial enrollment is required. In addition, multiple glucose-lowering drugs are available for the treatment of MASLD and T2DM, demonstrating efficacy in improving anthropometric and metabolic parameters, as well as improving fat and liver fibrosis. Turning to pharmacogenetics, it presents promising opportunities for personalized therapy in T2DM, but its clinical application is limited by insufficient replication studies and the relatively small effect sizes of identified genetic variants [120,121,122]. These studies also indicate potential relevance for MASLD treatment. However, no clinical trial data have yet been published that specifically evaluate the influence of genetic variants on the therapeutic response to GLP-1RA or SGLT2i in diabetic patients with MASLD. Further research is required to clarify this relationship.

## Figures and Tables

**Table 1:** Main non-invasive scores for estimating the liver fibrosis stage. NPV: negative predictive value. PPV: positive predictive value.

Score	Formula	Cut-Offs	Sensitivity	Specificity	Predictive Values
FIB-4 [31,46,47]	$\frac{Age(years)\times AST(U/L)}{Platelets(10^{9}/L)\times \surd ALT(U/L)}$	<1.45 → low risk	≈70% (rule-out), >80% (rule-in, high cut-off)	≈65–80% (depending on cut-off)	NPV: ~90% (<1.45) PPV: ~65–75% (>3.25)
		1.45–3.25 → indeterminate			
		>3.25 → high risk			
APRI [48,49]	$\frac{(AST/ULN\;AST)\times 100}{Platelets[10^{9}/L]}$	<0.5 → low risk	≈91 for low risk and 41 for advanced fibrosis	≈47 for low risk and 95% for advanced fibrosis	NPV high (86%) at a low cut-off (<0.5) PPV ~88% at >1.5
		0.5–1.5 → indeterminate			
		>1.5 → advanced fibrosis/cirrhosis			
NFS [50,51]	−1.675 + (0.037 × age) + (0.094 × BMI) + (1.13 × IFG/diabetes [yes = 1,no = 0]) + (0.99 × AST/ALT) − (0.013 × platelets) − (0.66 × albumin)	<−1.455 → low risk	≈77% (low cut-off, rule-out) ≈43% (high cut-off, rule-in)	≈71% (low cut-off) ≈96% (high cut-off)	NPV: ~88% (<−1.455) PPV: ~82% (>0.676)
		−1.455 to 0.676 → indeterminate			
		>0.676 → advanced fibrosis			

**Abbreviations**: FIB-4: fibrosis-4; AST: aspartate aminotransferase; ALT: alanine aminotransferase; NPV: negative predictive value; PPV: positive predictive value; APRI: AST-to-platelet ratio index; ULN: upper limit normal; NFS: Nonalcoholic Fatty Liver Disease Fibrosis Score; BMI: body mass index; IFG: impaired fasting glucose.

**Table 2:** Correspondence between liver elastometry values and the METAVIR score, according to the etiology of liver disease.

	Optimal Cut-Off for Significant Fibrosis (Stage ≥ F2)	Optimal Cut-Off for Advanced Fibrosis (Stage F3 or F4)	Optimal Cut-Off for Cirrhosis (Stage F4)
MASLD/MASH [52]		≥12 kPa	≥20 kPa
HBV [53]	>7 kPa	>8 kPa	>11 kPa
HCV [54]	7.1 kPa	9.5 kPa	12.5 kPa
Alcohol-related liver disease [55]	9 kPa	12.1 kPa	18.6 kPa
Primary biliary cholangitis [56]		9.28 kPa	15.2 kPa

**Abbreviations:** HBV: Hepatitis B virus; HCV: Hepatitis C virus; MASLD: Metabolic dysfunction–associated steatotic liver disease; MASH: Metabolic dysfunction-associated steatohepatitis.

**Table 3:** LSV cut-off based on probe type, based on the study by Oeda et al. [57].

Fibrosis	M Probe (kPa)	XL Probe (kPa)
Significant fibrosis (F2 or F3 or F4)	7	6.7
Advanced fibrosis (F3 or F4)	10.8	8.2
Cirrhosis (F4)	16.8	14.3

**Table 4:** Main drugs used in MASLD with their respective mechanisms of action, indications, side effects, and scientific evidence.

Agent	Mechanism of Action	Histologic/Hepatic Evidence	Additional Benefits	Main Adverse Effects	Indications/Restrictions
Resmetirom [85,86]	THR-β agonist: ↑ hepatic lipid metabolism	Phase 3 data: ↓ steatosis ↓ MASH in F2–F3 fibrosis	Targeted hepatic effect	Diarrhea, nausea	✓ F2–F3 MASH ✕ Cirrhosis [86,87]
GLP-1RA [88,89]	Incretin mimetics: ↑ satiety ↓ weight ↓ steatosis	LEAN: MASH resolution	Weight loss, ↓ CV, and renal risk	GI symptoms ↑ gallstones	✓ GLP-1RAs are safe to use in MASH (including compensated cirrhosis) and should be used for their respective indications (e.g., T2DM, obesity) [15] ✕ Personal or family history of Multiple Endocrine Neoplasia syndrome type 2 or medullary thyroid carcinoma [90]
SGLT2 inhibitors [15,91]	↓ renal glucose reabsorption: ↓ glucose, ↓ hepatic fat, osmotic diuresis	E-LIFT trial: ↓ hepatic fat (MRI-PDFF)	↓ CV and renal risk ↓ weight	Genitourinary infections, volume depletion	✓ SGLT2 can be used safely in MASLD and should be used for its respective indications (e.g., T2DM) [15] ✕ Renal impairment [92]
Pioglitazone [93,94]	PPAR-γ agonist: ↑ insulin sensitivity	PIVENS, other trials: ↑MASH and fibrosis	↑ glycemic control	↑ Weight heart failure bone fractures	✓ Pioglitazone is safe in biopsy-proven MASH + T2DM; ✕ Pioglitazone cannot be recommended as a MASH-targeted therapy [15] ✕ CHF ✕ Impaired kidney function [95]
Vitamin E (α-tocopherol) [93]	Antioxidant: ↓ oxidative stress	PIVENS: ↓ steatosis and inflammation	Oral intake	long-term CV risk, prostate cancer	Given the lack of robust histological efficacy, vitamin E cannot be recommended as a MASH-targeted therapy [15]
Metformin [96,97]	↑ insulin sensitivity via AMPK activation	No proven histologic benefit in MASH	Strong evidence of T2DM, ↓ metabolic risk	GI upset, lactic acidosis (rare)	✓ Metformin can be used safely in MASLD and should be used for its respective indications (e.g., T2DM) [15]
Tirzepatide [98]	Dual incretin agonists (GIP, GLP-1)	Phase 3: ↓weight, ↓ liver fat	In patients with T2D and inadequate glycemic control despite treatment with insulin glargine, the addition of subcutaneous tirzepatide improves glycemic control after 40 weeks	GI symptoms	✓ T2DM ✓ Obesity or overweight with at least one weight-related condition [99] ✓ Obstructive sleep apnea in adults with obesity [100]
Lanifibranor [101,102]	Pan-PPAR agonist	Phase 2b: ↓ MASH, ↓ fibrosis	Insulin sensitization	Weight gain, edema	Breakthrough therapy designation [103]

**Abbreviations:** THR-β: thyroid hormone receptor beta; MASH: metabolic dysfunction-associated steatohepatitis; GLP-1RA: glucagon-like peptide-1 receptor agonists; LEAN: liraglutide safety and efficacy in patients with non-alcoholic steatohepatitis; CV: cardiovascular; GI: gastrointestinal; MASLD: metabolic dysfunction–associated steatotic liver disease; T2DM: type 2 diabetes mellitus; E-LIFT: effect of empagliflozin on liver fat in patients with type 2 diabetes and nonalcoholic fatty liver disease; MRI-PDFF: magnetic resonance imaging–proton density fat fraction; PPAR-γ: peroxisome proliferator-activated receptor gamma; PIVENS: pioglitazone versus vitamin E versus placebo for the treatment of non-diabetic patients with nonalcoholic steatohepatitis; AMPK: 5′ adenosine monophosphate-activated protein kinase; GIP: gastric inhibitory polypeptide; and GLP-1: glucagon-like peptide-1. ✓ indicates the indications, ✕ indicates the restrictions. ↑ means increased. ↓ means decreased.

**Table 5:** Major randomized clinical trials with GLP-1RA, SGLT2i, and GIP/GLP-1 RA.

Clinical Trials Registry Number	Drug	Patients (*n*)	Primary Endpoints	Methods	Key Results
NCT01237119 (LEAN phase II) 2016 [88]	Liraglutide	52; liraglutide group (*n* = 26) and placebo group (*n* = 26)	MASH resolution without worsening of fibrosis after 48 weeks	Histologically proven	MASH resolution 39% in the liraglutide group vs. 9% the placebo group
NCT02970942 (phase II) 2021 [89]	Semaglutide	320; semaglutide 0.1 mg (*n* = 80), 0.2 mg (*n* = 78), or 0.4 mg (*n* = 82) or to receive placebo (*n* = 80)	MASH resolution without worsening of fibrosis after 72 weeks	Histologically proven	MASH resolution up to 59% in the semaglutide 0.4 mg vs. the placebo group, without worsening of fibrosis.
NCT04822181 (phase III) 2025 [105]	Semaglutide	800; semaglutide group (*n* = 534) and placebo group (*n* = 266)	MASH resolution without worsening of fibrosis after 72 weeks	Histologically proven	MASH resolution without worsening of fibrosis in 62.9% of patients in the semaglutide group vs. 34.3% in the placebo group.
NCT02970942 (phase II) 2024 [106]	Semaglutide	251 randomly assigned to receive once-daily s.c. semaglutide 0.1, 0.2, or 0.4 mg	MASH resolution without worsening of fibrosis after 72 weeks	Histologically proven	MASH resolution without worsening of fibrosis was significantly higher in patients receiving semaglutide 0.4 mg (58.5%) than in those receiving placebo (22.0%).
NCT03987451 (phase II) 2023 [107]	Semaglutide	71 randomly assigned (2:1) to receive either once-weekly subcutaneous semaglutide 2.4 mg or a visually matching placebo	Improvement in liver fibrosis of one stage or more without worsening of MASH after 48 weeks	Histologically proven	No significant improvement in liver fibrosis of one stage or more without worsening of MASH.
NCT04166773 (phase II) 2025 [108]	Tirzepatide	190 randomly assigned to receive tirzepatide (5, 10, or 15 mg) or placebo once weekly	MASH resolution without worsening of fibrosis	Histologically proven	MASH resolution and fibrosis improvement were associated with body weight reduction, improved glycemic control, and normalization of liver fat.
NCT03131687 (phase II) 2020 [109]	Tirzepatide	316 received either once weekly tirzepatide (1, 5, 10, or 15 mg), dulaglutide (1.5 mg), or placebo for 26 weeks	Effect of tirzepatide on biomarkers of MASH and fibrosis	Biomarkers	Significant improvement of MASH-related biomarkers in a T2DM population.
IRCT20190122042450N1 2020 [110]	Empagliflozin	90 randomly assigned to empagliflozin 10 mg/day (*n* = 43) or placebo (*n* = 47)	Change in controlled attenuation parameter from baseline to 24 weeks of treatment. The secondary endpoint was the change in liver stiffness values from baseline to 24 weeks	Transient elastography with controlled attenuation parameter	No significant difference in controlled attenuation parameter score was observed between the two groups. Liver stiffness values significantly decreased after 24 weeks in the empagliflozin group.
UMIN000022155 2019 [111]	Dapaglifozin	57 randomly assigned to a dapagliflozin group with a dose of 5 mg/d (*n* = 33) or a control group (*n* = 24)	Change in controlled attenuation parameter from baseline to 24 weeks of treatment. The key secondary endpoint was the change in LSV from baseline to 24 weeks of treatment,	Transient elastography with controlled attenuation parameter	Significantly decreased controlled attenuation parameter score and improvement of liver fibrosis only in patients with significant liver fibrosis.

Abbreviations: LEAN: liraglutide safety and efficacy in patients with non-alcoholic steatohepatitis; and MASH: metabolic dysfunction-associated steatohepatitis.

## Data Availability

Data supporting reported results can be found in published articles available in PubMed.

## Abbreviations

The following abbreviations are used in this manuscript:

NASH	Nonalcoholic steatohepatitis
NAFLD	Nonalcoholic fatty liver disease
MAFLD	Metabolic dysfunction-associated fatty liver disease
MASLD	Metabolic dysfunction-associated steatotic liver disease
MASH	Metabolic dysfunction-associated steatohepatitis
CAP	Controlled Attenuation Parameter
MRI-PDFF	Magnetic resonance imaging–proton density fat fraction
T2DM	Type 2 diabetes mellitus
FLI	Fatty liver index
MetALD	Metabolic dysfunction-associated steatotic liver disease and Alcohol-related liver disease
EASL–EASD–EASO	European Association for the Study of the Liver, European Association for the Study of Diabetes, European Association for the Study of Obesity
GLP-1RAs	Glucagon-like peptide-1 receptor agonists
SGLT-2i	Sodium–Glucose Co-transporter Inhibitors
ROS	Reactive oxygen species
LPS	Lipopolysaccharide
TLR	Toll-like receptors
HCC	hepatocellular carcinoma
MACE	major adverse cardiovascular events
ADA/EASD	American Diabetes Association/European Association for the Study of Diabetes
FIB-4	Fibrosis-4
NFS	Nonalcoholic Fatty Liver Disease Fibrosis Score
VCTE	vibration-controlled transient elastography
MRE	magnetic resonance elastography
APRI	AST-to-platelet ratio index
LSM	Liver stiffness measurement
LSV	Liver stiffness value
HbA1c	Hemoglobin A1c
MRI	Magnetic resonance imaging
DPP-4	Dipeptidyl peptidase-4
SGLT2	Sodium glucose co-transporter-2
GLP-1	Glucagon-like peptide-1
